# Apoptin‐derived peptide reverses cisplatin resistance in gastric cancer through the PI3K–AKT signaling pathway

**DOI:** 10.1002/cam4.1380

**Published:** 2018-03-09

**Authors:** Danyang Zhou, Wenjing Liu, Songhe Liang, Banghao Sun, Anqi Liu, Zhongqi Cui, Xue Han, Lijie Yuan

**Affiliations:** ^1^ Department of Biochemistry and Molecular Biology Harbin Medical University Daqing Campus Daqing Heilongjiang 163319 China; ^2^ Clinical Laboratory of Daqing People’s Hospital Daqig Helongjiang 163310 China; ^3^ Department of Clinical Laboratory Medicine Shanghai Tenth People's Hospital of Tongji University Shanghai 200072 China

**Keywords:** Apoptin‐derived peptide, cisplatin resistance, gastric cancer, PI3K/AKT/ARNT

## Abstract

The prognosis of gastric cancer (GC) remains poor due to clinical drug resistance, and novel drugs are urgently needed. Apoptin‐derived peptide (AdP) is an antitumor polypeptide constructed in our laboratory that has been used to combat cisplatin (CDDP) resistance in GC cells. MTT and colony‐formation assays and Hoechst 33342 staining were used to measure the cytotoxicity of CDDP and AdP in GC cells. Cell apoptosis was measured using an Annexin‐V‐FITC/PI dual staining assay. Western blot analysis was conducted to detect the expression of proteins in the PI3K/AKT signaling pathway and resistance‐related markers. AdP exerted a specific cytotoxic effect on GC cells and CDDP‐resistant GC cells in a concentration‐ and time‐dependent manner. AdP also suppressed cell invasion and migration. Additionally, AdP inhibited the expression of p85, AKT, p‐p85, p‐AKT, multidrug resistance 1 (MDR1), and aryl hydrocarbon nuclear translocator (ARNT) in the PI3K/AKT/ARNT signaling pathway, which promoted apoptosis and necrosis in GC cells. AdP promoted apoptosis in CDDP‐resistant GC cells by suppressing the PI3K/AKT/ARNT signaling pathway and might be considered a candidate agent for the clinical treatment of cisplatin‐resistant GC.

## Introduction

Gastric cancer (GC) is the third major cause of global cancer‐related death 1, 2. Because of its high incidence and high mortality, GC is a serious threat to humans 3, 4, and China is among the nations with the highest incidence of GC 5. The most frequently used chemotherapeutic agent for GC treatment is cisplatin (CDDP) 6. Based on phase III trials in Japan, a combination of CDDP and the 5‐fluorouracil‐related drug S‐1 has been considered the first‐line chemotherapy treatment for advanced GC 7. However, the overall efficacy of CDDP treatment is limited in the clinic due to the development of drug resistance 8. Therefore, identifying new targets involved in drug resistance may foster the development of new strategies for improving chemotherapy targeting GC.

The molecular mechanisms of drug resistance are complex and involve antiapoptosis 9, 10, drug metabolism, and drug efflux mechanisms 11. One of the main mechanisms of CDDP resistance is the escape of tumor cells from apoptosis 12, 13. The PI3K/AKT pathway has been considered a target for overcoming acquired anticancer resistance 14, and AKT is one of the key multidrug resistance genes 15. The aryl hydrocarbon receptor (AHR) and aryl hydrocarbon nuclear translocator (ARNT) (also known as hypoxia‐inducible factor (HIF)‐1β) are a member of the basic helix‐loop‐helix PER/AHR/ARNT/SIM (bHLH‐PAS) family of transcription factors 16. Under normoxic conditions, ARNT serves as a dimerization partner for several transcription factors and multidrug resistance 1 (MDR1), which contributes to tumorigenesis and drug resistance 17, 18, 19, 20.

Our laboratory constructed an apoptin‐derived peptide (AdP) as an antitumor polypeptide that was originally designed based on the structure of the apoptosis hormone. Our previous study showed that AdP has anticancer activities in vitro and in vivo by promoting apoptosis and inhibiting metastasis 21. In addition, we found that AdP inhibits MMP‐9 expression through inactivation of PI3K/AKT/mTOR signaling 22. However, no data regarding AdP and drug‐resistant GC are available.

Apoptin‐derived peptide contains an SH3 domain, and it binds specifically to PI3K, thereby inhibiting tumor cell growth. Our preliminary results show that AdP down‐regulates the expression of p85‐mediated PI3K/AKT signaling pathways. In addition, p85 is a PI3K subunit, and AdP decreases the expression of p85, thus inhibiting its phosphorylation, ultimately leading to the decreased expression of pathway proteins. Therefore, AdP may preserve the antitumor properties of CDDP‐resistant GC cells, and phosphorylated p85 might be a target for the treatment of gastric cancer.

## Materials and Methods

### Cell lines, compounds, and reagents

Human GC cell line SGC‐7901 (cisplatin‐sensitive GC cells) and SGC‐7901/CDDP (cisplatin‐resistance GC cells) were obtained from ShangHai Qiao Du Biotechnology Co.Ltd, ShangHai, China; the MGC‐803 (cisplatin‐sensitive GC cells) and SW‐620 (colon cancer cells) were obtained from the Department of the Harbin Medical University, and the human glioma cell lines U87‐MG and U251‐MG were obtained from the Department of the Third Affiliated Hospital of Harbin Medical University. Human embryonic kidney (HEK) 293 cells were obtained from the Shanghai Institutes for Biological Sciences Cell Resource Center. SGC‐7901, SGC‐7901/CDDP, and MGC‐803 were routinely cultured in RPMI‐1640 medium (GE Healthcare Life Sciences, Logan, UT), and HEK293, U87, and U251 were cultured in DMEM (Dulbecco's modified Eagle's medium, GE Healthcare Life Sciences) in a humidified cell incubator with an atmosphere of 5% CO_2_ at 37°C until passing by trypsinization after reaching 80–90% confluence. The culture media were supplemented with 15% fetal bovine serum (FBS; Zhejiang Tianhang Biological Technology Co.Ltd, Zhejiang, China) and 1% penicillin–streptomycin solution (Beyotime Biotechnology, Shanghai, China). SGC‐7901/CDDP was cultured with its maximum cisplatin resistance concentration 1 μg/mL.

### Western blot analysis

Total protein was extracted from cells after treatment with AdP (40 μg/mL, 60 μg/mL, 80 μg/mL, or 100 μg/mL) for 24 h. The proteins were separated and transferred onto a polyvinylidene difluoride (PVDF) membrane (Millipore, Billerica, MA) for immunoblotting as previously described. The membranes were incubated with primary antibodies against AKT (1:300; Boster, Wuhan, China), p‐AKT (1:500; Rui Ying Biological, Suzhou, China), phosphoinositide 3‐kinase (p85) (1:500; Rui Ying Biological), p‐p85 (1:300; Rui Ying Biological), aryl hydrocarbon receptor nuclear translocator (ARNT) (1:500; Boster), multidrug resistance 1 (MDR1) (1:300; Boster), and β‐actin (1:5000; ZhongshanGoldenbridge Biotechnology, Beijing, China). Then, the membranes were incubated with secondary antibody (ZhongshanGoldenbridge Biotechnology). Finally, the membranes were developed using the chemiluminescence method.

### Real‐time PCR

Total RNA was extracted from cultured cells using TRIzol Reagent (Invitrogen, Carlsbad, CA) and reverse‐transcribed with the Transcriptor First Strand cDNA synthesis kit (Haigene, Shenzhen,China), following the manufacturer's instructions. Quantitative real‐time PCR was performed on an iCycleriQ Real‐Time PCR Detection System using iQ SYBR Green Supermix (Haigene). Real‐time PCR data were quantified with the Bio‐Rad iCycler system software and are expressed as relative mRNA expression normalized to a value of 1.0 for the unstimulated control group. Control reactions without cDNA were performed as a negative control. Fold change was calculated taking the mean of the controls as the baseline. Each sample was analyzed at least in triplicate. β‐actin was used as an internal control. The forward and reverse primers used are listed in Table 1.

**Table 1:** Primer sequences of genes

PI3K	5′‐CCACGACCATCATCAGGTGAA‐3′	5′‐CCTCACGGAGGCATTCTAAAGT‐3′
AKT	5′‐ACGATGAATGAGGTGTCTGT‐3	5′‐TCTGCTACGGTGAAGTTGTT‐3′
β‐actin	5′‐CCTGTACGCCAACACAGTGC‐3′	5′‐ATACTCCTGCTTGCTGATCC‐3′

### MTT assay

Cell viability of human embryonic kidney (HEK) 293 cells, **t**he human GC cell line SGC‐7901 (cisplatin‐sensitive GC cells) and SGC‐7901/CDDP (cisplatin‐resistance GC cells) and MGC‐803 (cisplatin‐sensitive GC cells) and SW‐620 (colon cancer cells), the human glioma cell lines U87‐MG and U251‐MG, cell viability were assessed using an MTT assay (Amersco, Ohio). Cells at a density of 1~2 × 10^4^ were seeded into 96‐well culture plates and incubated for 18 h with AdP at concentrations of 10 μg/mL and 70 μg/mL, among them SW‐620 with AdP at concentrations of 10 μg/mL and 80 μg/mL, U87, U251 cells with AdP at concentrations of 20 μg/mL and 200 μg/mL, respectively, for 24 h or SGC‐7901, SGC‐7901, HEK 293 cells with AdP at a concentration of 60 μg/mL for 4 h, 6 h, 8 h, 12 h, 18 h, and 24 h. Then, the cells were incubated with 20 μL MTT reagent (5 mg/mL) at 37°C for 4 h. The formazan product was dissolved in 150 μL DMSO at room temperature for 10 min, and the optical density (OD) was read on a spectrophotometer (ELx 808; Gene Company Limited, Shenyang, China) at 490 nm. HEK 293/SGC‐7901 and SGC‐7901/CDDP cells were cultured in serum‐free DMEM/1640 medium as control group.

### Plate cloning experiment

SGC‐7901 and SGC‐7901/CDDP cells were seeded into the six‐well culture plate at 10^3^ cells/well, and cultured in 15% FBS medium containing RPMI culture medium for a period of 14 days, then the colonies were fixed with 4% paraformaldehyde and stained with 1% crystal violet, and then washed with PBS, finally, observed by camera.

### Immunohistochemical staining

Immunostaining was performed using the SP kit (Zhongshan Goldenbridge Biotechnology). Wax seal tissue slices were dewaxed, rehydrated, and then boiled in a 0.01 mol/L citrate buffer for 15 min. The activity of endogenous peroxidase was blocked by a hydrogen peroxide of 0.3% for a period of 10 min. The samples were incubated with rabbit anti‐p‐AKT (1:50; Rui Ying Biological, Suzhou, China), p85 (1:50; Rui Ying Biological), p‐p85 (1:50; Rui Ying Biological), ARNT (1:50; Boster, Wuhan, China), MDR1 (1:50; Boster) antibodies, mouse anti‐AKT (1:100; Boster), or HSP70 (1:50; Beyotime Biotechnology) antibody at 4°C overnight. Then, the samples were incubated with the secondary antibody at room temperature for 1 h. The immune reaction was revealed by a DAB (3,3′‐diaminobenzidine) kit. After stained with hematoxylin, the tissue preparations were dehydrated and mounted. Sections exposed to nonimmune sera were used as negative controls. The staining score is as follows: 0, no staining; 1+, minimum staining; 2+, moderate to strong staining of at least 20% of cells; 3+, strong staining of at least 50% of cells. 0 or 1+ stained cases were classified as negative, and those with 2+ or 3+ staining were considered positive.

### HE staining

Hematoxylin–eosin (HE) staining was performed on the slices which were dewaxed with xylene and rehydrated through graded ethanol series. After hematoxylin staining for 5 min, hydrochloric acid alcohol 2 sec, after rinsing with water for 15 min, and then stained with Yihong 1 min, and then dehydrated by gradient alcohol and xylene, and finally with a sealed mirror.

### Hoechst staining

Hoechst 33342 dyes (Beyotime Biotechnology) are cell permeable nucleic acid stains, which are useful for the recognition of DNA damage and cell apoptosis by monitoring the emission spectral shifts of the dyes. Cells were stained with Hoechst 33342 (10 μg/mL) in PBS at room temperature for 20 min, and then washed to remove unbound dyes. Observation and photography were performed using a fluorescence microscope (Nikon eclipse TE2000‐S, Sendai, Japan).

### AO/EB staining

Acridine orange (AO) can penetrate through the membrane integrity of normal cells and incorporate into the nuclear DNA to emit bright green fluorescence. Bromine Bromium (EB) can only penetrate the damaged cells through the cell membrane, embedded in nuclear DNA to emit red fluorescence. The cells treated with AdP (30 μg/mL and 60 μg/mL), PBS (control group), or cisplatin (CDDP) 1 μg/mL for 24 h were digested by trypsin (Beyotime Biotechnology). The cell suspension was collected and incubated with 10 μL of AO (Nachuan Biological, Haerbin, China) solution (100 μg/mL), and 10 μL of EB (100 μg/mL) was added to the suspension for staining at room temperature for 5 min. Samples were examined with a Live Cell Workstation (Leica AF6000, Wetzlar, Germany).

### Cell apoptosis assay and cell cycle analysis

Apoptosis of SGC‐7901 and SGC‐7901/CDDP cells was detected using an Annexin‐V‐propidium iodide (PI) apoptosis detection kit (Beyotime Biotechnology) according to the manufacturer's instructions. Briefly, cells were resuspended in 1× binding buffer at 1 million cells/mL. Then, 5 μL Annexin‐V‐FITC antibody, 10 μL PI, and 195 μL Annexin‐V‐FITC combined liquid were added to the cell suspension under dark conditions at room temperature for 15 min. The preparations were analyzed using flow cytometry (BD Accuri C6, Franklin Lake).

### Cell invasion and migration assays

Cells at a density of 2 × 10^5^ were seeded into 12‐well culture plates and incubated for 18 h with AdP at concentrations of 10 μg/mL, 20 μg/mL, and 30 μg/mL; after 48 h, cells were washed three times with PBS and fixed in 4 % paraformaldehyde for 15 min. Then, cells were stained with Hoechst 33342 (10 μg/mL) in PBS at room temperature for 20 min, and then washed to remove unbound dyes. Observation and photography were performed using a fluorescence microscope (Nikon eclipse TE2000‐S, Sendai, Japan).

### Clinical database

Samples of human gastric cancer and cisplatin‐resistant gastric cancer tissues were collected from the Fifth Affiliated Hospital of the Harbin Medical University. Of 10 samples, five were from patients with effective treatment by chemotherapy and five from those with no obvious effects after two courses of the same chemotherapy. The specific information of the patients is shown in Table 2.

**Table 2:** Gastric cancer patient information

Variables	Gastric cancer		Gastric cancer resistance	
	Number	%	Number	%
Age (years)				
≥59	4	40	3	30
<59	1	10	2	20
Sex				
Male	3	30	3	30
Female	2	20	2	20
Classification				
High differentiated	2	20	5	50
Medium differentiation	3	30	0	0
Tumor type				
Adenoma cancer	5	50	3	30
Undifferentiated cancer	0	0	2	20
Chemotherapy effect				
Improve	5	50	0	0
No effect	0	0	5	50

Expression of PI3K/AKT mRNA and protein expression in human GC PI3K/AKT protein expression in GC tissues and normal tissues was determined from the human protein atlas (http://www.proteinatlas.org). GC PI3K/AKT gene expression was determined through analysis of Roessler and TCGA databases, which are available through cBioPortal for Cancer Genomics (http://www.cbioportal.org/). High and low groups were defined as above and below the mean, respectively.

### Statistical analysis

Statistical analysis of data was performed using SPSS version 13.0 for Windows (SPSS Inc, Chicago, IL). Data are presented as mean ± SE; Statistical comparisons among multiple were performed using ANOVA with a Holms–Sidak post hoc test and *P *≤* *0.05 was considered statistically significant.

## Results

### Structural features of apoptin‐derived peptide

**Figure 1:**
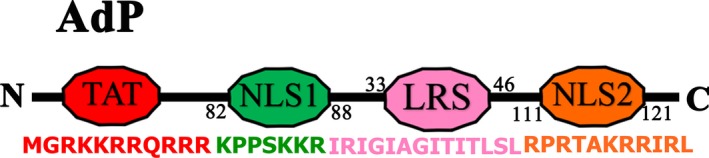
Diagram of the structure of AdP.

Apoptin‐derived peptide is an apoptin‐based polypeptide that was based on apoptin and contains 43 amino acids that correspond to the nuclear localization sequence (NLS1, amino acids 82–88), NLS2 (amino acids 111–121), and leucine‐rich sequence (LRS) structural domains (amino acids 33–46) and is enriched with hydrophobic amino acids. The molecular weight of AdP is 5.2 kDa 23. The structure of AdP is shown in Fig. 1.

### Inhibitory effects of AdP on viability of tumor cells

**Figure 2:**
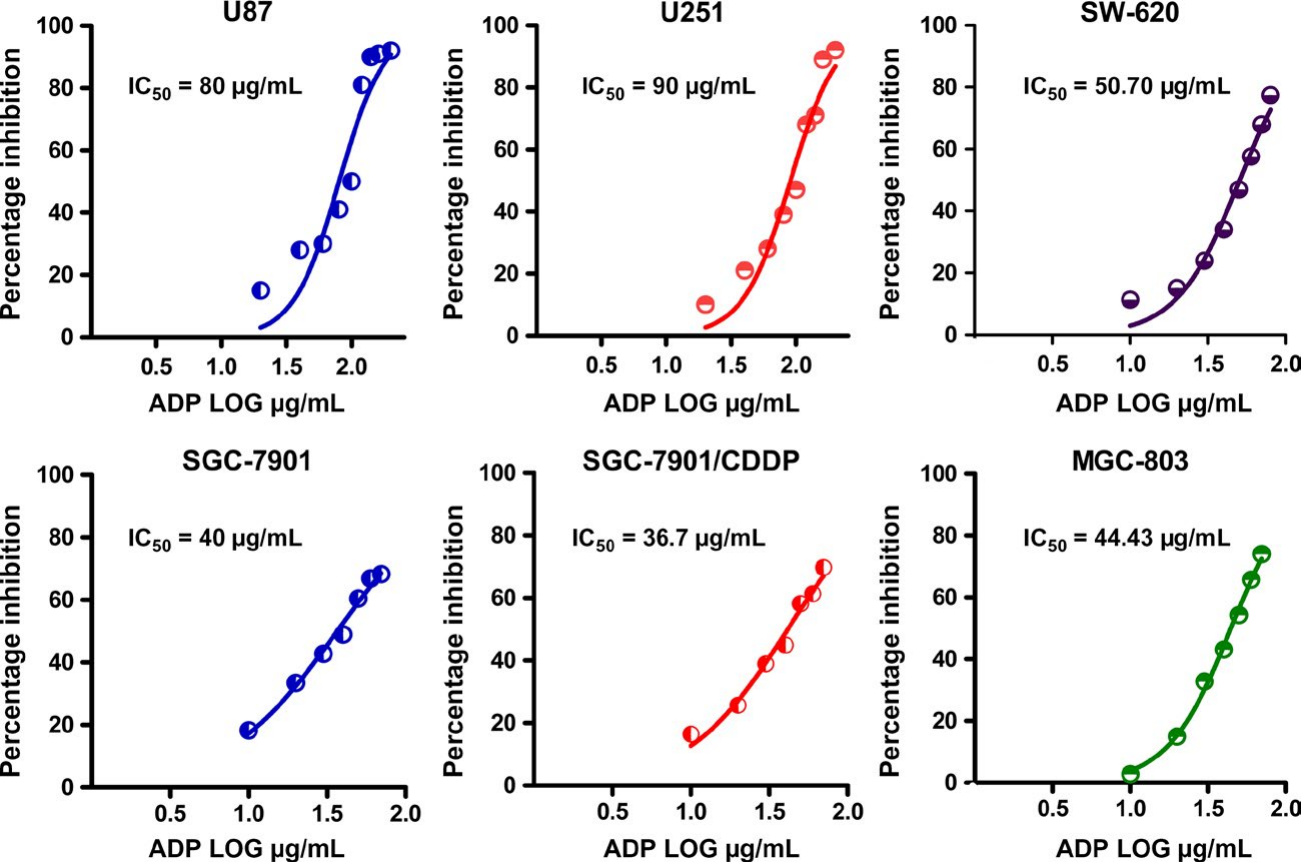
Based on MTT experiments, the IC_50_ values of AdP were 40 ± 0.23 μg/mL (SGC‐7901), 36.7 ± 0.41 μg/mL (SGC‐7901/CDDP), 44.43 μg/mL (MGC‐803), 50.7 μg/mL (SW‐620), 80 μg/mL (U87), and 90 μg/mL (U251) at 24 h.

We tested the effects of varying forms of AdP on the viability of several selected tumor cells, including glioma, colon cancer, and GC cells, using an MTT assay. As illustrated in Fig. 2, AdP inhibited cell viability in a concentration‐dependent manner in both CDDP‐sensitive and CDDP‐resistant GC cells. The IC_50_ values at 24 h were as follows: SGC‐7901 GC cells, 40 μg/mL; SGC‐7901/CDDP GC cells, 36.7 μg/mL; MGC‐803 cells, 44.43 μg/mL; SW‐620 cells, 50.7 μg/mL; U87 cells, 80 μg/mL; and U251 cells, 90 μg/mL. We found that AdP had an excellent inhibitory effect on GC cells, and CDDP‐resistant GC cells were sensitive to AdP.

### Proapoptotic action of AdP on gastric cancer cells

**Figure 3:**
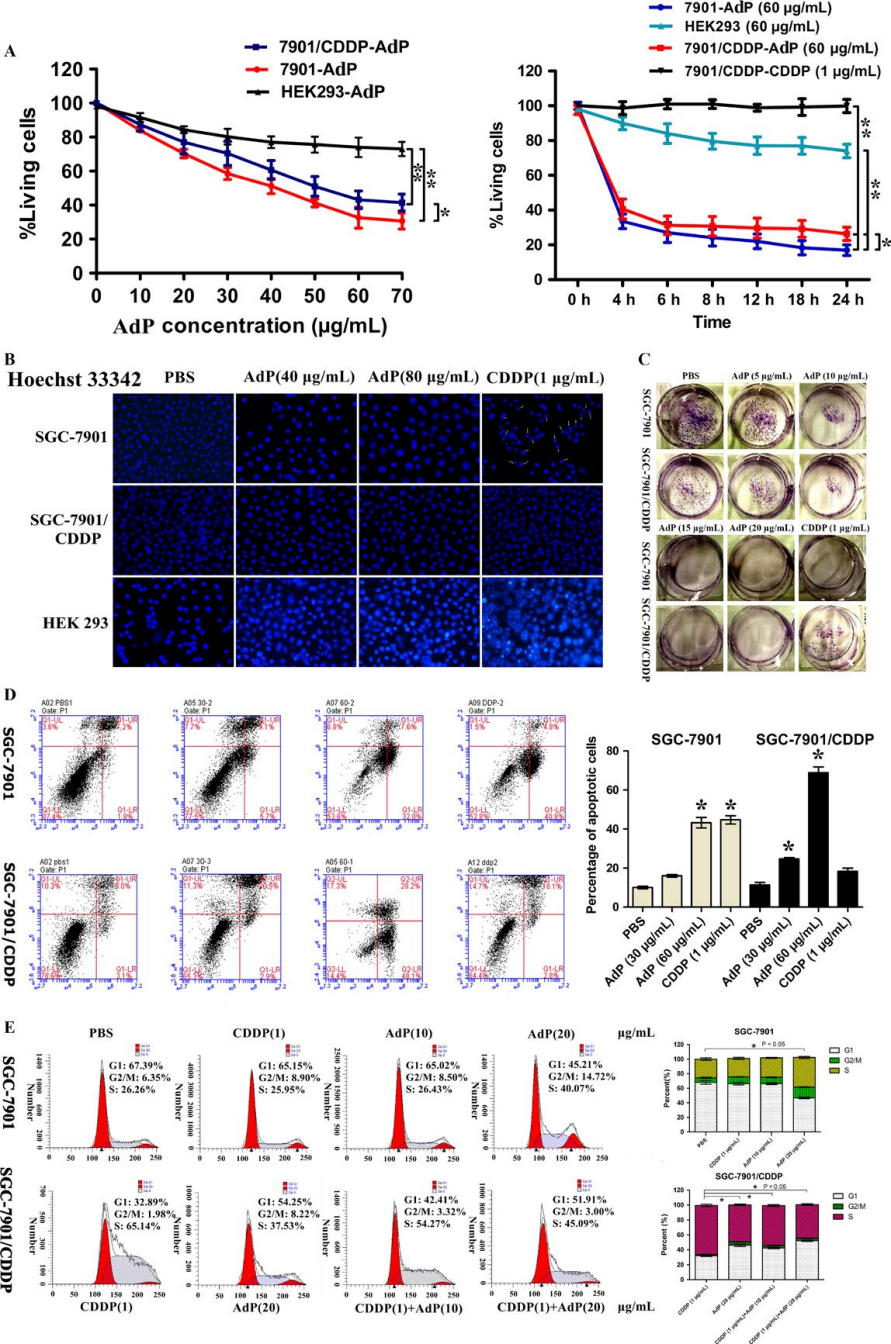
Comparative efficacies of AdP on inhibition of GC cell growth, and the effect of AdP on cell viability. (A) An MTT assay shows the concentration‐ and time‐response effects of AdP on cell viability. The data are presented as the mean ± standard deviation of three independent experiments, *P <* *0.05, **P <* *0.01. (B) Hoechst 33342 dye staining of nuclei of S3GC‐7901, SGC‐7901/CDDP, and HEK293 cells after a 24‐h treatment (scale bar, 4 μm). PBS, AdP (40 μg/mL), AdP (80 μg/mL), CDDP (1 μg/mL). (C) Plate cloning experiment. The AdP concentration gradient ranged from 5 μg/mL to 20 μg/mL. When treated with 15 μg/mL AdP, the colony‐forming ability of the two cell lines reached the lowest point. (D) SGC‐7901 and SGC‐7901/CDDP cells were treated with 30 μg/mL and 60 μg/mL AdP for 24 h, stained with Annexin‐V and propidium iodide and then subjected to flow cytometry. The data are presented as the mean ± standard deviation of three independent experiments, *P *<* *0.05.(E) ADP leads to S phase reduction, and G2/M phase increased in SGC‐7901 as same as SGC‐7901/CDDP, thereby inducing apoptosis, *P *<* *0.05.

SGC‐7901/CDDP cells were treated with 1 μg/mL CDDP to evaluate the cytotoxic effects of AdP. MTT assay results showed that CDDP had no significant influence on the survival of SGC‐7901/CDDP cells, supporting the CDDP‐resistant property of this cell line, as previously described 22. In sharp contrast, AdP suppressed cell viability in a dose‐dependent and time‐dependent manner (Fig. 3A). Treatment with AdP at 60 μg/mL for 6 h produced large effects in both SGC‐7901 and SGC‐7901/CDDP cells. However, AdP had no obvious effect on immortalized human embryonic kidney (HEK293) cells (Fig. 3A). We observed chromatin condensation in SGC‐7901 and SGC‐7901/CDDP cells using Hoechst staining, which indicated a reduction in cell viability that was attributable to apoptotic cell death. This observation was also supported by the morphological changes in the cells, which became round and fragmented. Similar effects were not observed in HEK293 cells (Fig. 3B).

Furthermore, we determined whether AdP treatment could affect the colony‐forming ability of SGC‐7901 and SGC‐7901/CDDP cells. When treated with 15 μg/mL AdP, the colony‐forming ability of these two cell lines was substantially weakened (Fig. 3C). CDDP (1 μg/mL) induced a similar inhibition in SGC‐7901 cells but had no significant inhibitory effect on SGC‐7901/CDDP cells.

Annexin‐V‐FITC staining and flow cytometry were performed to evaluate the level of apoptosis in SGC‐7901 and SGC‐7901/CDDP cells 24 h after AdP treatment. CDDP was used as a positive control, and PBS was used as a negative control. As depicted in Fig. 3D, AdP treatment induced SGC‐7901 cell apoptosis in a concentration‐dependent manner. CDDP treatment of SGC‐7901 cells caused 45.6% apoptosis (Fig. 3D). By comparison, in the SGC‐7901/CDDP group, CDDP (1 μg/mL) produced only approximately 20.9% apoptosis after a 24‐h treatment. In contrast, AdP induced considerable apoptotic cell death in SGC‐7901/CDDP cells in a strong time‐ and dose‐dependent fashion. These results suggest that AdP increased the CDDP sensitivity of SGC‐7901/CDDP cells and induced apoptosis.

To evaluate the effect of AdP on cell cycle arrest, we detected the cell cycle distribution of SGC‐7901/CDDP and SGC‐7901cells treated with different concentrations of AdP (10 μg/mL, 20 μg/mL) for 24 h. The proportion of GC cells in the G2/M phase increased. These results suggest that AdP can increase the G2/M phase population, thereby leading to apoptosis, and the AdP‐treated SGC‐7901/CDDP cells have an increased sensitivity to CDDP.

### Overexpression of p‐AKT, MDR1, and ARNT in drug‐resistant GC cells

**Figure 4:**
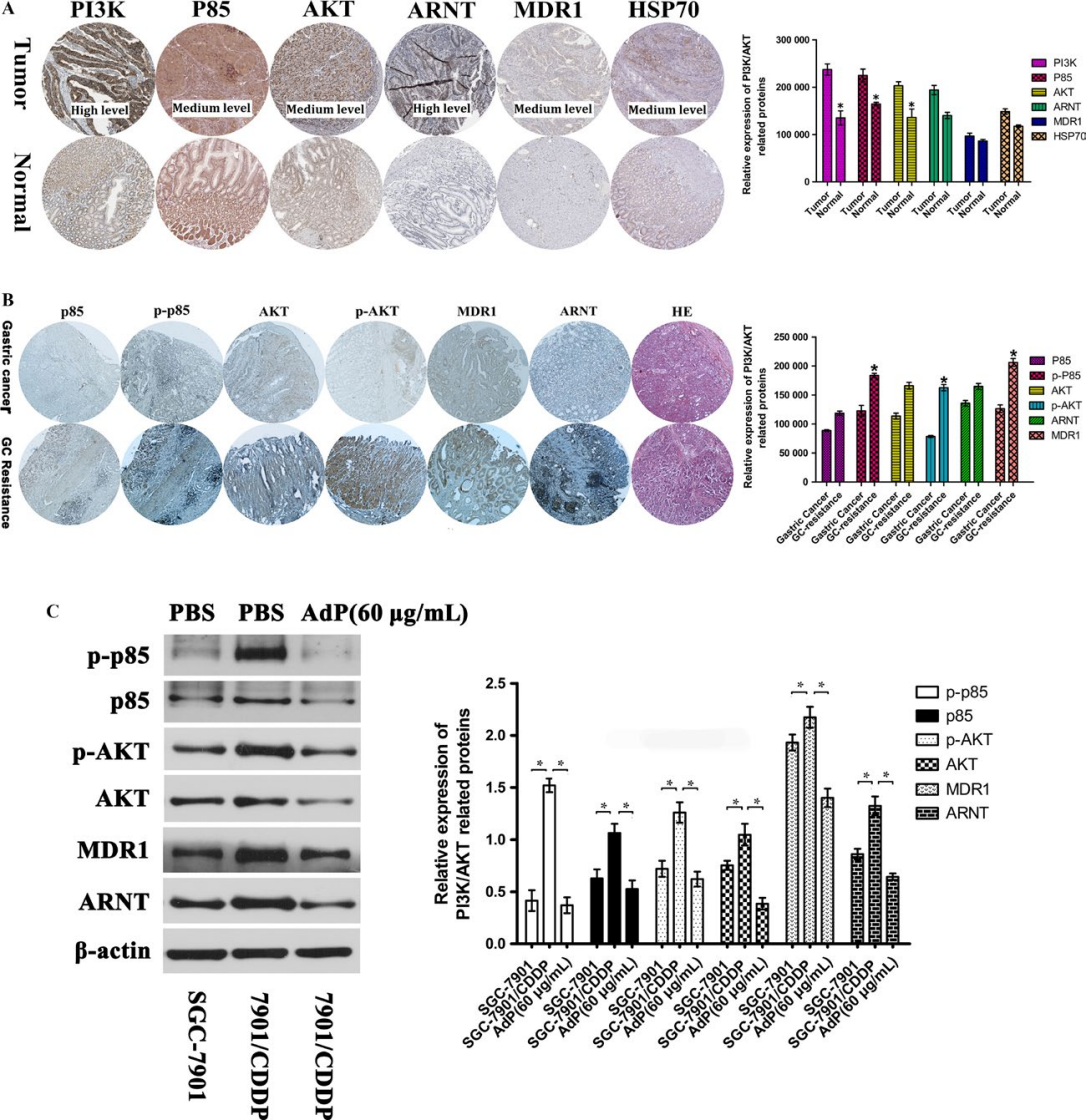
PI3K, p‐p85, p‐AKT, ARNT, and HSP70 are overexpressed in human gastric cancer (GC) specimens; p‐AKT, MDR1 and ARNT are highly expressed in platinum‐resistant GC tissues. (A) PI3K, AKT, ARNT, MDR1, and HSP70 expression in normal stomach tissue and GC specimens. Images were taken from the Human Protein Atlas (http://www.proteinatlas.org) online database. (B) p‐AKT, MDR1, and ARNT expression in platinum‐resistant GC tissues was determined by immunohistochemical analysis (*n* = 5); scale bar, 50 μm; and *P *<* *0.05 versus the control. (C) The expression levels of p‐p85, p85, p‐AKT, AKT, ARNT, and MDR1 in SGC‐7901, SGC‐7901/CDDP cells and with AdP (60 μg/mL) in SGC‐7901/CDDP cells were determined by immunoblotting.

To determine the clinical relevance of the expression of the above proteins, we first analyzed their expression in clinical specimens in the human protein atlas (http://www.proteinatlas.org). GC cells exhibited positive expression of p‐AKT, MDR1, and ARNT proteins, and normal stomach tissues had negative or weak expression of these proteins. In particular, the expression of PI3K and ARNT in GC tissues was significantly higher than that in normal stomach tissues (Fig. 4A). To verify whether elevated p‐AKT, MDR1, and ARNT expression confer CDDP resistance in GC cells, we first determined the protein levels of p‐AKT, MDR1, and ARNT in GC and CDDP‐resistant GC tissues relative to that in normal gastric epithelium. An immunohistochemistry (IHC) analysis was performed to measure the expression of p‐AKT, MDR1, and ARNT in a series of wax‐sealed human platinum‐resistant GC samples (Fig. 4B). The results revealed that the expression of these proteins in platinum‐resistant GC tissues was higher than that in normal GC tissues. As shown in Fig. 4C, at the cellular level, we found that p‐p85, p85, p‐AKT, AKT, ARNT, and MDR1 were highly expressed in SGC‐7901/CDDP cells. However, the expression of these proteins was significantly down‐regulated by AdP at a concentration 60 μg/mL. Our results showed a reasonably good correlation between the expression of these proteins and CDDP resistance for most of the GC cell lines examined.

### Inhibition of the PI3K/AKT/ARNT pathway by AdP

**Figure 5:**
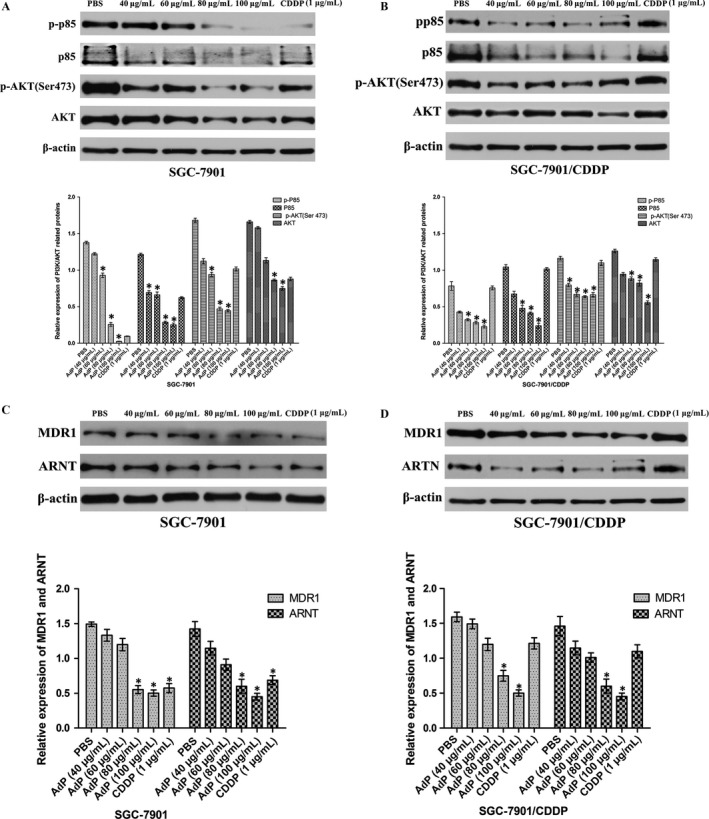
Apoptin‐derived peptide through the PI3K/AKT pathway to inhibit the proliferation of SGC‐7901/ CDDP cells. (a), (b) SGC‐7901 and SGC‐7901/CDDP cells were treated with AdP for the concentration gradient, and p‐p85, p85, p‐AKT, AKT were determined by immunoblotting assay. (c), (d) SGC‐7901 and SGC‐7901/CDDP cells were treated with AdP for the concentration gradient, and ARNT, MDR1 were determined by immunoblotting assay; *P *<* *0.05.

Activation of PI3K was observed in nearly half of the human GC cells 24. We found that SGC‐7901/CDDP cells expressed higher levels of phosphorylated AKT (p‐AKT) and phosphorylated p85 (p‐p85). Similar results were observed in human GC tissues and drug‐resistant GC tissues; of the 10 patient samples, five patients had been clinically diagnosed with gastric adenocarcinoma, and five had two courses of GC chemotherapy (Table 2) without significant improvement and had associated metastatic tissue. AdP at concentrations from 40 μg/mL to 100 μg/mL significantly down‐regulated the phosphorylation of p85 (p‐p85) and AKT (p‐AKT) proteins. AdP at the same concentrations significantly reduced the total protein levels p85 and AKT. In addition, AdP also significantly reduced the levels of p‐AKT, p‐p85, AKT, and p85 in SGC‐7901 cells (Fig. 5A,B).

P‐glycoprotein (P‐gp), encoded by the MDR1 gene, is the classical multidrug resistance protein 25, and it functions as an ATP‐dependent drug efflux pump that reduces the intracellular concentrations of chemotherapeutic agents, including CDDP 26. Our results indicated that MDR1 expression was down‐regulated in AdP‐treated SGC‐7901/CDDP cells.

ARNT protein expression is controlled by PI3K signaling. Our results showed that ARNT expression was markedly down‐regulated in SGC‐7901/CDDP cells (Fig. 5C,D).

**Figure 6:**
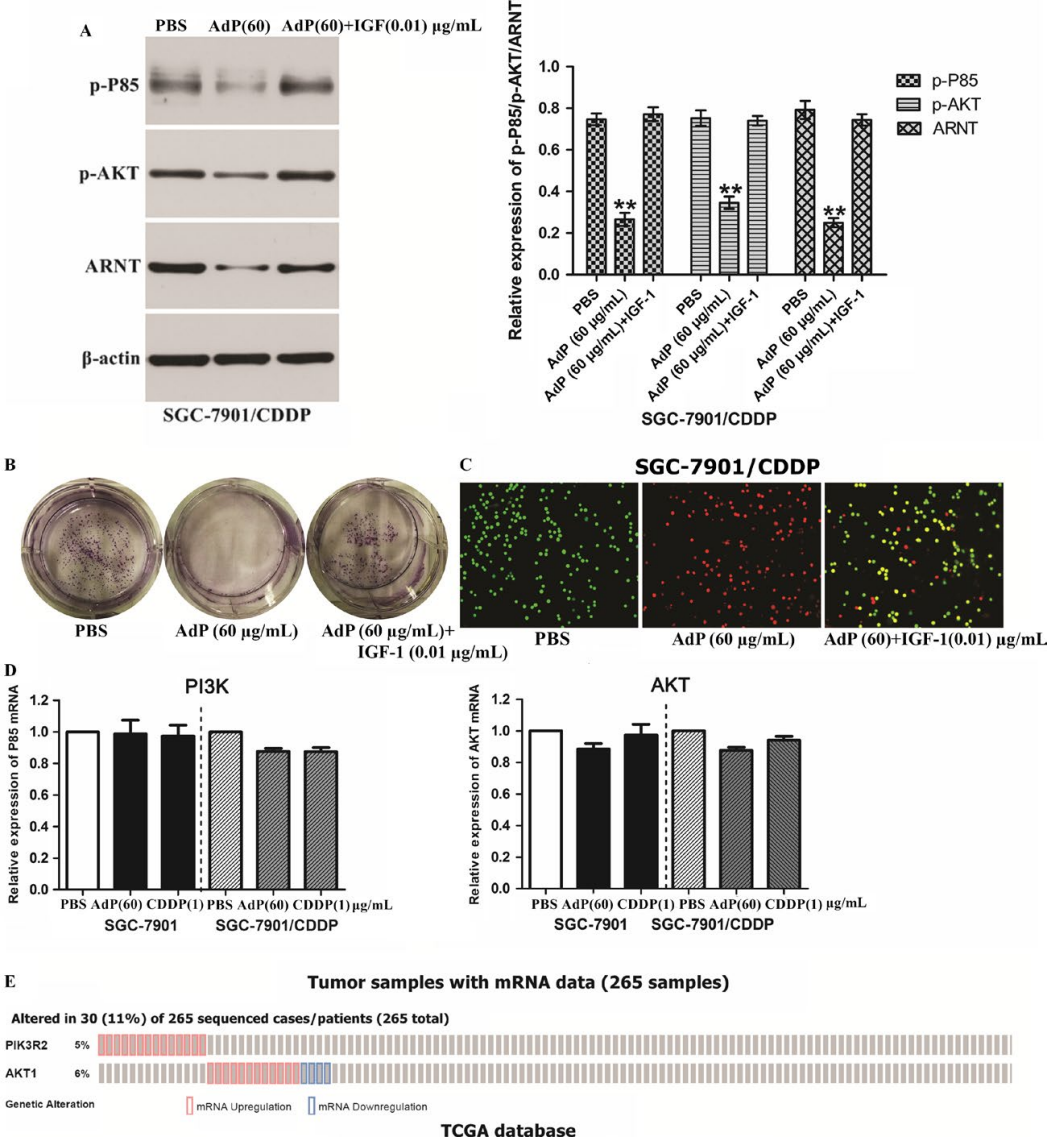
Apoptin‐derived peptide treatment correlated with ARNT by PI3K/AKT signaling pathway. (A) SGC‐7901/CDDP cells were treated with AdP (60 μg/mL) for 24 h. Then, p‐p85, p‐AKT, ARNT expression was determined by immunoblotting assay; *P *<* *0.05. (B, C) Plate cloning and AO/EB. SGC‐7901/CDDP cells were treated with PBS, AdP (60 μg/mL), AdP (60 μg/mL)+IGF (10 ng/mL)‐1 for 24 h. Then, observation was performed with a live cell workstation. (D) qRT‐PCR. p85 and AKT expression was not markedly changed in the presence of the AdP compared with that in the control group. (E) PI3K, AKT mRNA expression in the TCGA gastric cancer RNAseq (IlluminaHiSeq; *n* = 265) data set.

Apoptin‐derived peptide significantly decreased PI3K activity and ARNT expression (Fig. 6A). Activation of AKT, a downstream component of the PI3K pathway, is observed in many GC cells and tissues 27. We predicted that AdP might regulate ARNT protein levels by inhibiting the PI3K/AKT signaling pathway. To examine this notion, we used SCG‐7901/CDDP cells treated with CDDP (1 μg/mL) with or without addition of AdP (60 μg/mL) or the PI3K activator IGF‐1 (T&L Biological Technology, Beijing, China) (10 ng/mL) plus AdP for 24 h. Our Western blotting results demonstrated that IGF‐1 and AdP cotreatment did not have a significant inhibitory effect on PI3K/AKT or ARNT. AdP treatment alone notably decreased p85 and AKT activities and down‐regulated ARNT expression. The results of plate cloning experiments and AO/EB double staining provided further evidence in support of the role of AdP in inhibiting the PI3K/AKT signaling pathway (Fig. 6B,C). Addition of both the PI3K activator IGF‐1 and AdP promoted SGC‐7901/CDDP cell proliferation and significantly weakened apoptosis, and addition of AdP alone produced almost no viable cells. Collectively, these results revealed that AdP promoted ARNT down‐regulation by inhibiting the PI3K/AKT signaling pathway to enhance CDDP resistance.

Figure 6D shows that the mRNA levels of p85 and AKT, as determined by qRT‐PCR, were not altered by AdP. We further found that the mRNA levels of PI3K and AKT were significantly altered in only 30 human clinical samples of 265 patients from the TCGA database (Fig. 6E). This finding suggests that GC is associated with a low level of PI3K and AKT mRNA expression.

### Inhibitory effect of AdP on the invasion and migration of gastric cancer cells

**Figure 7:**
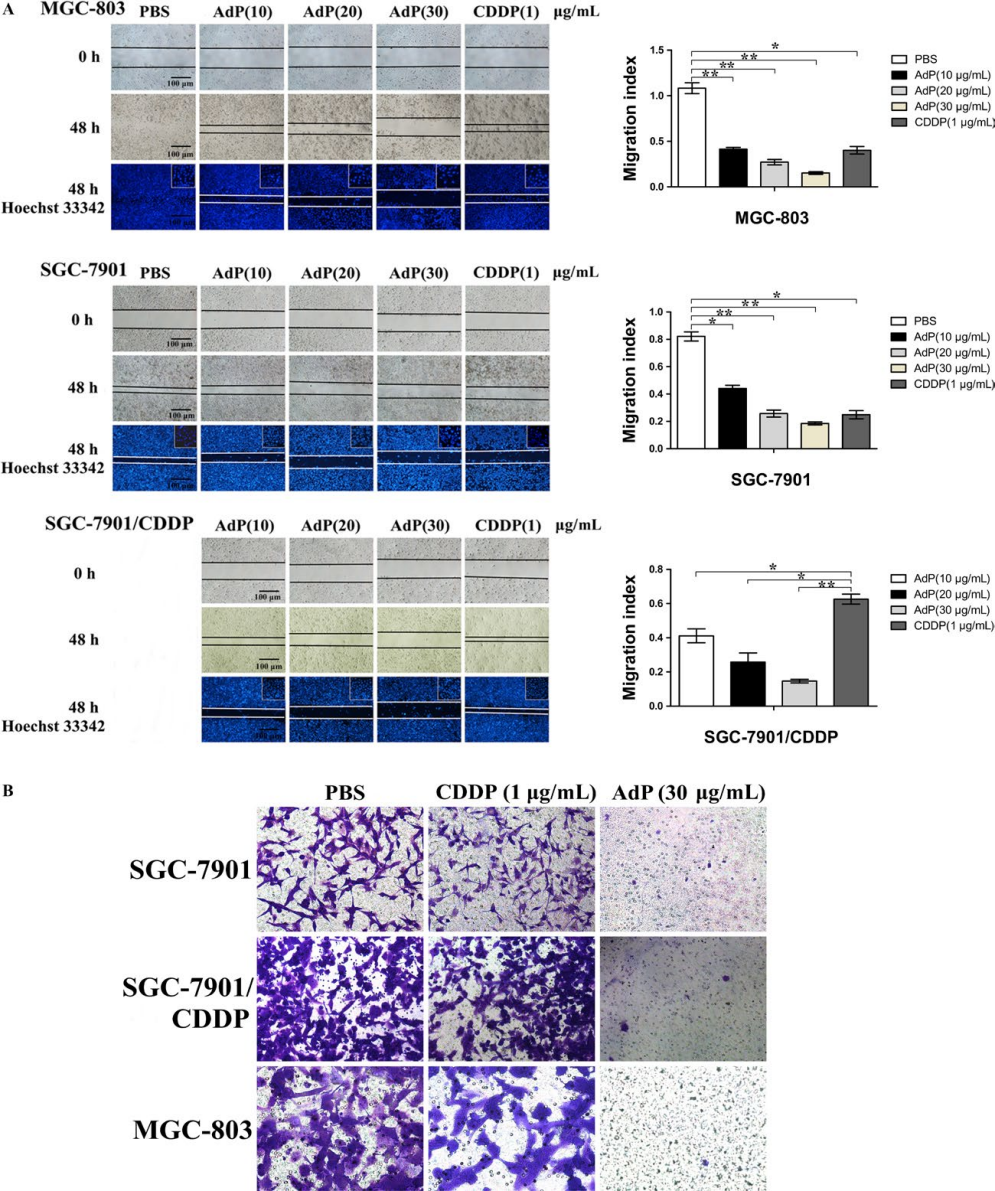
Inhibitor effect of AdP on invasion and migration of gastric cancer cells. (A) Cells were treated with various concentrations of AdP (10 μg/mL, 20 μg/mL and 30 μg/mL) or PBS for 48 h, and the migrating cells were visualized by phase‐contrast imaging. The results are expressed as the mean±SEM of at least three independent experiments. *P <* *0.05, **P <* *0.01 compared with the control group. (B) Cells were treated with AdP (30 μg/mL), CDDP (1 μg/mL), or vehicle for 24 h, and migrating cells were visualized by phase‐contrast imaging.

Different concentrations of AdP were applied to MGC‐803, SGC‐7901, and SGC‐7901/CDDP cells, and PBS was used as a positive control. Then, invasion and migration experiments were performed. The results showed that AdP significantly reduced the number of invasive cells and effectively inhibited the migration of GC cells. As shown in Fig. 7, AdP significantly inhibited the migration ability of MGC‐803, SGC‐7901, and SGC‐7901/CDDP cells in a dose‐dependent manner. Our results suggest that application of AdP significantly inhibits the migration and invasion of human GC cells in a dose‐dependent manner.

## Discussion

Apoptin is a functional protein encoded by the chicken anemia virus (CAV) vp3 gene that can specifically induce tumor cell apoptosis without affecting normal somatic cells 28. In our previous studies, we found that AdP exerts a strong inhibitory effect on glioma cells, especially on the invasion and migration of these cells 23. GC cells and glioma cells both have a similarly strong ability to invade and migrate, and the acquired drug resistance in GC cells is closely associated with the activation of the PI3K/AKT signaling pathway 22. It is known that AdP suppresses glioma cell invasion and migration via suppressing PI3K/AKT signaling 22. Therefore, we proposed that AdP might be effective in treating gastric cancer and abrogate the drug resistance of GC through inactivating PI3K/AKT signaling. The present study indeed generated the data in support of our hypothesis. These data suggest that AdP exerted a significant proapoptotic effect on CDDP‐resistant GC at the cellular level.

As pivotal oncogenes, p‐p85 and p‐AKT, the components of the PI3K/AKT pathway, have been shown to participate in tumorigenesis, and to confer poor clinical outcomes 29. We analyzed clinical drug‐resistant GC samples using immunohistochemical analysis, and the results showed that p‐p85 and p‐AKT expression in drug‐resistant GC tissues were significantly increased, and no significant differences in the expression of the apoptosis‐associated and CDDP metabolism‐related proteins were observed between CDDP chemotherapy‐resistant and nonresistant GC tissues. These results led us to focus our study on the conventional PI3K/AKT/ARNT mechanism of drug resistance mediated by drug transportation. Various studies have shown that the PI3K/AKT signaling pathway is associated with tumor resistance to chemotherapy and radiotherapy. The published results show that a combination of AKT inhibitors improves the sensitivity of cancer cells to chemotherapy and radiotherapy 30, 31, suggesting that the PI3K/AKT signaling pathway is closely related to GC resistance to CDDP.

**Figure 8:**
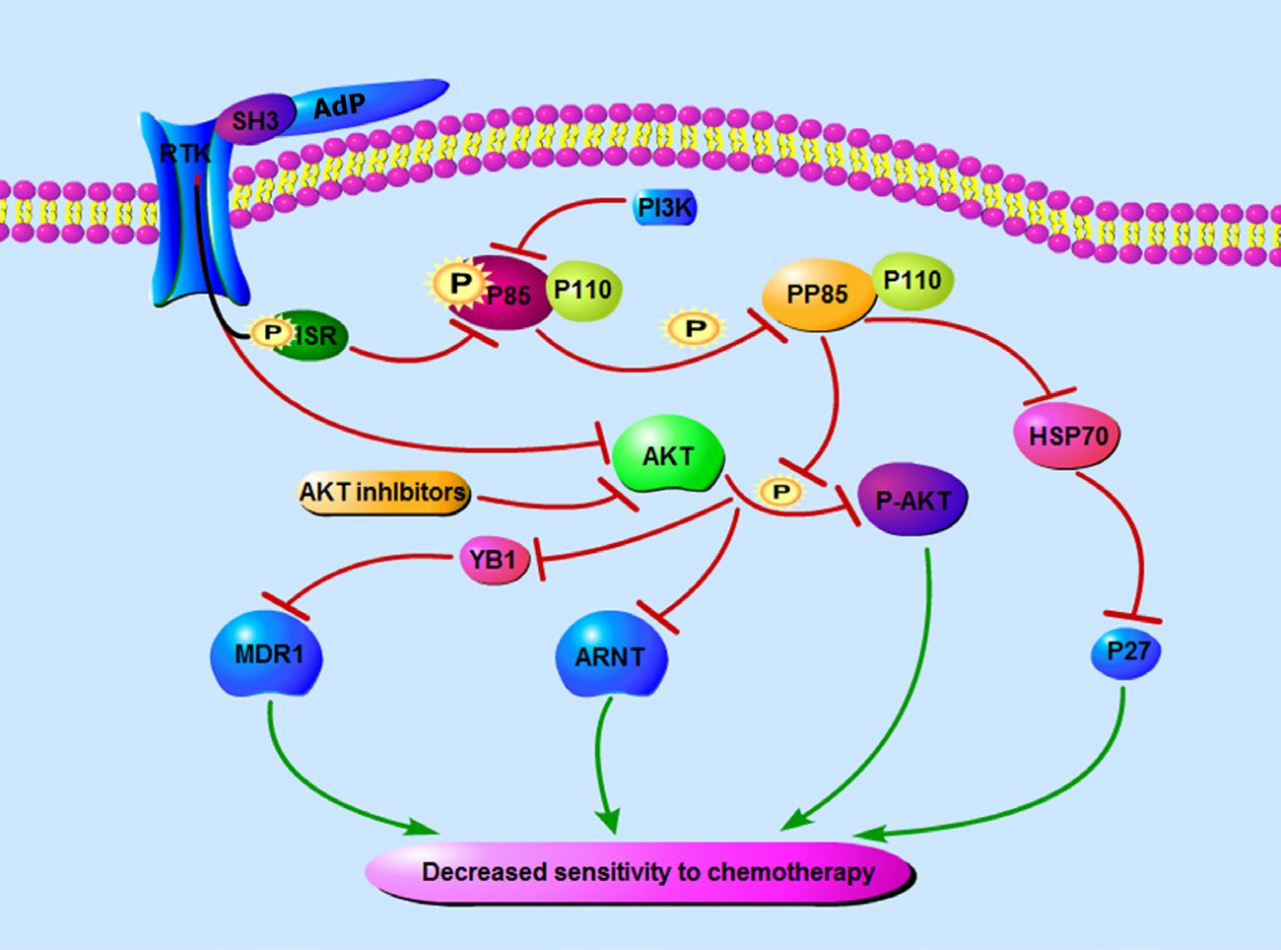
Inhibitory effect of AdP on SGC‐7901/CDDP cell PI3K/AKT signaling pathway.

AdP has an SH3‐binding domain that can bind RTK receptors to inhibit p85 phosphorylation, which in turn inhibits AKT phosphorylation (p‐AKT). We initially envisaged that AdP containing the SH3 domain could inhibit p‐p85 and p‐AKT (Fig. 8). This study indicated that AdP simultaneously down‐regulated the expression of p85 and AKT and suppressed their activation to inhibit the PI3K/AKT signaling pathway, thereby increasing chemotherapy sensitivity. This increase in chemotherapy sensitivity occurred not only in SGC‐7901/CDDP cells but also in SGC‐7901 cells.

Abnormal expression of drug transporters such as MDR1 is a major cause of chemotherapy failure 32. Increased expression of MDR1 in GC has been reported to be associated with poor prognosis and multidrug resistance 33, 34. We found that MDR1 was involved in resistance to CDDP in GC cells, and AdP down‐regulated MDR1 expression, thereby increasing the sensitivity of GC cells to CDDP. Strikingly, one study reported that AKT activity decreases the suppression of YB1 phosphorylation, reducing MDR1 expression, which can promote CDDP sensitivity in GC cells 35. Therefore, we have reason to speculate that by inhibiting the PI3K/AKT pathway, AdP further inhibits YB1 phosphorylation and reduces MDR1 expression to increase the sensitivity of GC cells to CDDP.

Under normoxic conditions, ARNT, also known as HIF‐1β, serves as a dimerization partner for several transcription factors, including MDR1, thereby contributing to tumorigenesis and drug resistance 16, 19, 20. The results of this study show that ARNT expression in CDDP‐resistant GC cells and tissues was higher than that in GC cells and tissues. Additionally, AdP strongly down‐regulated ARNT expression, and this down‐regulation was achieved by inhibiting AKT phosphorylation. The following results confirmed our conclusions. With the addition of the PI3K activator IGF‐1R, AdP down‐regulated the expression of ARNT and diminished the ability of AdP to inhibit the viability of SGC‐7901/CDDP cells. These results suggest that AdP inhibits the PI3K/AKT/ARNT signaling pathway to induce apoptosis of SGC‐7901/CDDP cells and enhance sensitivity to CDDP.

A study by Jaganmohan R and team showed that an apoptotic apoptin‐derived 10 aa peptide (aa: 81–90) had an ideal cytotoxic effect on imatinib‐resistant leukemia cells and could be used as a potential therapeutic drug for leukemia 36. We designed that AdP with an LRS, which provides a flexible connection between the NLSs. Additionally, the N‐terminus of the peptide chain was tagged with a TAT sequence. We know that NLS1 has a SH3 domain that can induce apoptosis through the PI3K/AKT signaling pathway. In the previous experiment, we screened the APC/C1‐binding domain (amino acids: 82–121) by gel blocking assay (Electrophoretic mobility shift assay, EMSA), which contained NLS1 and NLS2 37. They can induce tumor cell cycle arrest, which induced apoptosis. We found that AdP can reduce the S phase of SGC‐7901/CDDP cells and inhibit cell proliferation. Thus, we hypothesized that AdP had a better effect on CDDP‐resistant GC. The LRS domain has a β‐corner secondary structure, which largely protects the spatial structure and activity of NLS1 and NLS2, and the LRS domain is rich in leucine. Our previous studies showed that the LRS domain interacts with AKT and can inhibit AKT phosphorylation. In conclusion, we previously designed a synthetic apoptin‐derived peptide, which superimposes the functions of NLS1, NLS2, and LRS domain, while maintaining the relative space and activity of the three domains continuously maintained by the LRS domain. Each structure of AdP has important significance for apoptosis. Therefore, it is a potential treatment for reversing GC resistance.

Development of novel chemo‐sensitive antitumor drugs for clinical application is of great importance 38. Here, we evaluated the effects of AdP and present several novel features that suggest the therapeutic efficacy of AdP in GC. First, AdP had a specific cytotoxic effect on GC cells and CDDP‐resistant GC cells and promoted apoptosis and necrosis in a concentration‐ and time‐dependent manner. Second, AdP inhibited the expression of p85 and AKT at the post‐transcriptional level, and reduced the levels of p‐p85 and p‐AKT. Third, a survey of clinical samples indicated that p‐p85, p‐AKT, MDR1, and ARNT expressions were positively correlated with cisplatin‐resistant GC and that AdP down‐regulated the expression of these proteins to promote GC cell sensitivity to cisplatin. Finally, through its SH3‐binding domain, AdP inhibited PI3K/AKT/ARNT signal transduction and promoted GC cells apoptosis. Therefore, this study suggests that AdP may be a novel therapeutic agent for the clinical treatment of GC. This study provides a theoretical basis for the development of new drugs for GC and presents evidence that supports the benefits of the clinical application of AdP.

## Conflict of Interest

None declared.

## Ethical approval

This article does not contain any studies with human participants or animals performed by any of the authors. /All applicable international, national, and/or institutional guidelines for the care and use of animals were followed. /All procedures performed in studies involving human participants were in accordance with the ethical standards of the institutional and/or national research committee and with the 1964 Helsinki declaration and its later amendments or comparable ethical standards.

## Informed consent

For this type of study, formal consent is not required. Informed consent was obtained from all individual participants included in the study.
