# Correction to apoptin‐derived peptide reverses cisplatin resistance in gastric cancer through the PI3K–AKT signaling pathway

**DOI:** 10.1002/cam4.70056

**Published:** 2024-08-02

Zhou D, Liu W, Liang S, et al. Apoptin‐derived peptide reverses cisplatin resistance in gastric cancer through the PI3K–AKT signaling pathway. Cancer Med 2018 Apr;7(4):1369–1383. doi: 10.1002/cam4.1380. Epub 2018 Mar 9.

Figure 5: One error is that PP85 in Figure 5B and ARNT in Figure 5D use the same image. In fact, the strip of PP85 was used incorrectly. Another error is Figure 5A and Figure 5B applied the same β‐actin. We have found the correct image for correction. Figure 5A and 5B was corrected.

In the annotation of Figure 5D, “ARNT” was mistakenly labeled as “ARTN.”

Figure 4 and Figure 6: Figure 4C and Figure 6A were suspected of using the same β‐actin. In order to prove the accuracy of the results, we conducted repeated experiments. Figure 4C and Figure 6A were corrected.

As for Figure 6B, there is also an error in the composition diagram; the repeated test is also carried out. Figure 6B was corrected.

The corrected figures are shown below.

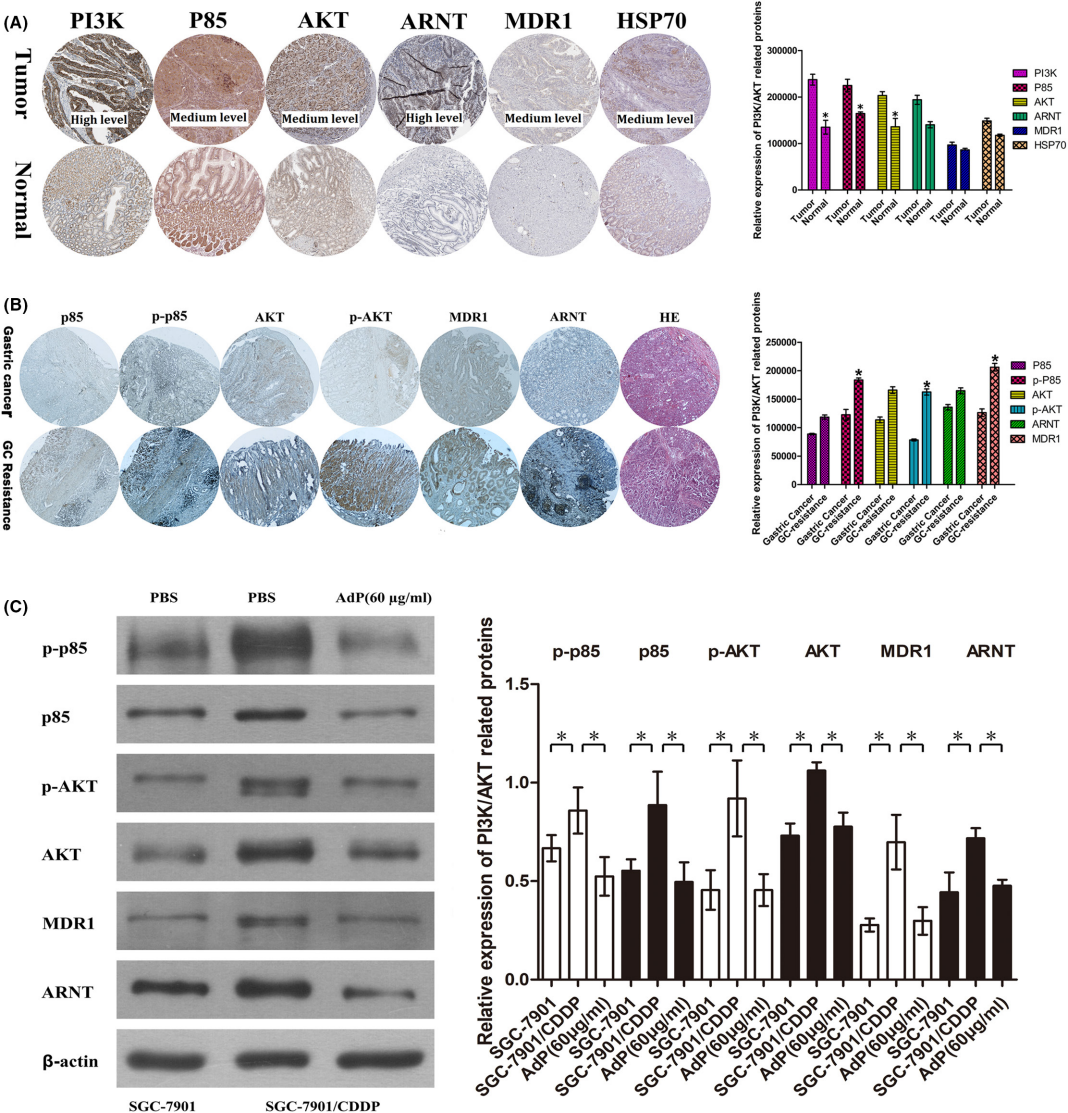

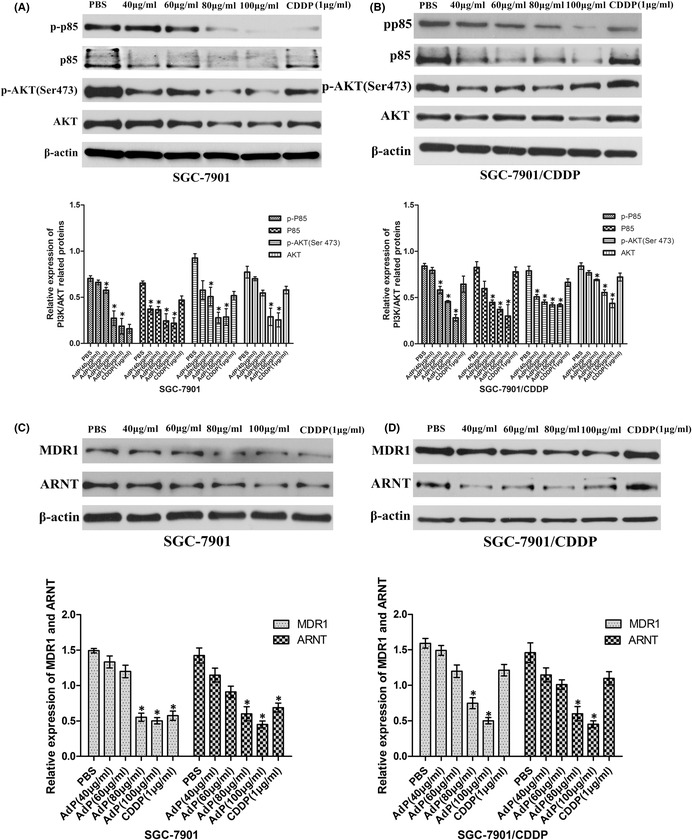

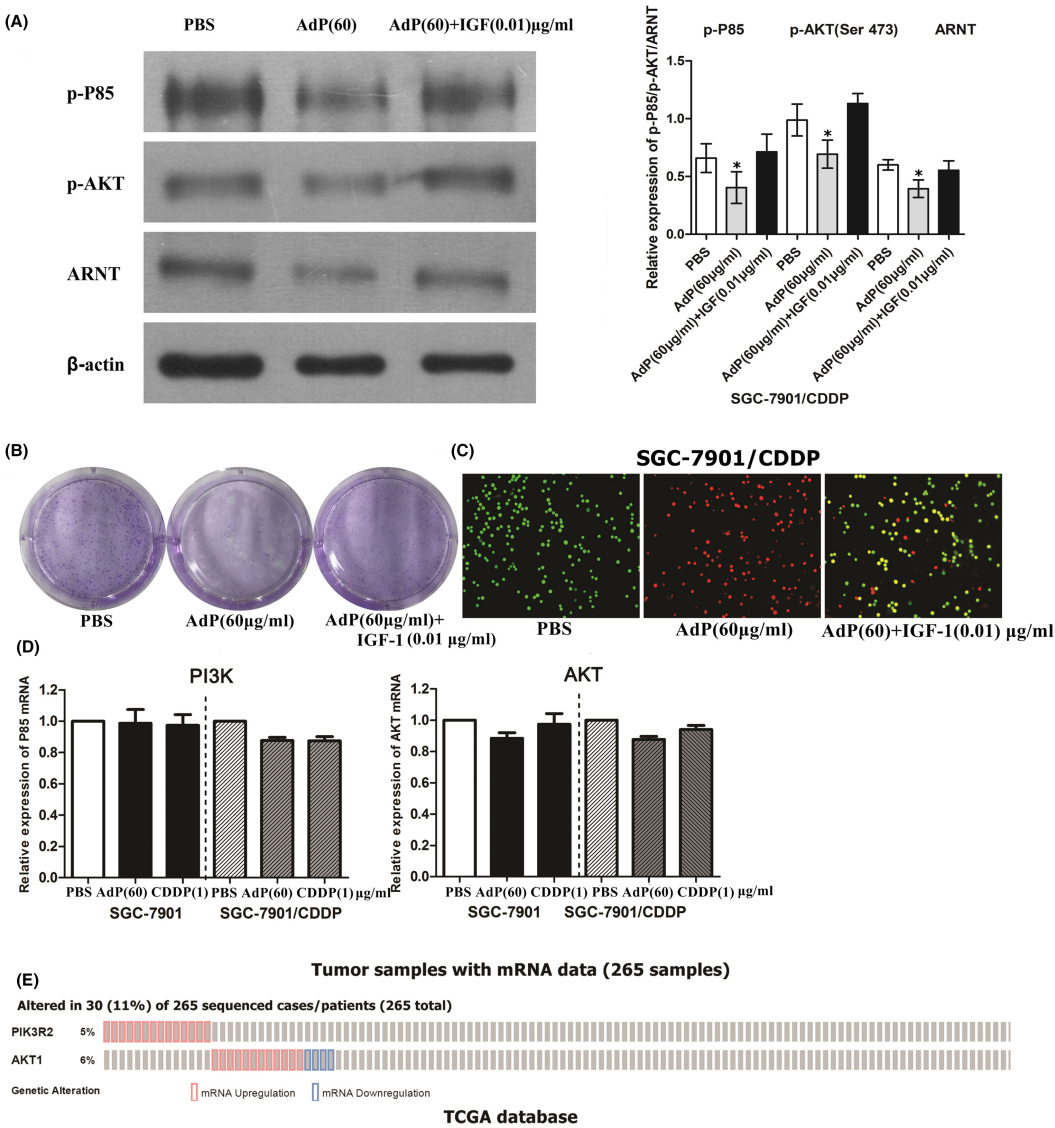

We apologize for these errors.

